# A lower canal fill rate and unreestablished vertical femoral offset may increase the risk of the postoperative periprosthetic fractures after cementless bipolar hemiarthroplasty for femoral neck fractures in elderly patients

**DOI:** 10.1371/journal.pone.0285789

**Published:** 2023-05-19

**Authors:** Xiaoxiao Zhou, Houlin Ji, Jiajun Wu, Haixiao Chen, Yang Yang

**Affiliations:** 1 Department of Orthopedics, Shanghai University of Medicine & Health Sciences Affiliated Zhoupu Hospital, Shanghai, China; 2 Jinji Lake Community Health Service Center of Suzhou Industrial Park, 215000, Jiangsu, China; 3 Department of Orthopedics, Taizhou Hospital of Zhejiang Province, Affiliated to Wenzhou Medical University, Zhejiang, China; Sohag University Faculty of Medicine, EGYPT

## Abstract

**Background:**

Periprosthetic fractures (PPFs) is one of the major causes of failure of hip arthroplasty with cementless stem; however, studies on the incidence and risk factors of PPFs after cementless hemiarthroplasty for femoral neck fractures (FNFs) are lacking.

**Methods:**

This retrospective study included patients who underwent cementless bipolar hemiarthroplasty for displaced intracapsular FNFs. The demographic data were reviewed, Dorr classification was used to describe morphology of the femur, radiological parameters were measured including stem-shaft angle, canal fill ratio (CFR), canal flare index (CFI), morphologic cortical index (MCI), canal calcar ratio (CCR), and vertical and horizontal femoral offset.

**Results:**

The sample comprised 10 men and 46 women (affected hip: left, 38; right, 18). The mean patient age was 82.82±10.61 (range, 69–93) years, and the mean hemiarthroplasty to PPFs time was 26.28±14.04 (range, 6.54–47.77) months. Seven (12.28%) patients had PPFs. A significant relationship was found between the incidence of PPF and CFR (p = 0.012), patients had a significantly smaller femoral stem CFR (0.76%±0.11%) than controls (0.85%±0.09%). The PPFs group had a significant shorter and unreestablished vertical femoral offset (p = 0.048).

**Conclusions:**

A smaller femoral stem CFR associated with a potentially unacceptably high PPFs risk in uncemented hemiarthroplasty for displaced FNFs may result from mismatched prosthesis and bone dimensions in the elderly population, especially when accompanied by a poorly reestablished vertical femoral offset. With increasing evidence of the benefits of cemented fixation, a cemented stem for the treatment of displaced intracapsular FNFs is recommended for such a elderly frail population.

## Introduction

Bipolar hemiarthroplasty has become a common treatment for displaced intracapsular femoral neck fractures (FNFs) in the elderly. Opinions among orthopedic surgeons regarding prosthesis choice in the elderly remain divided. Some surgeons prefer the use of cementless stems because cemented stems carry the risk of increased operative time and perioperative mortality secondary to fat and marrow emboli [1].

A recent multicenter randomized controlled study showed that cemented hemiarthroplasty was associated with a lower rate of PPFs and better quality of life compared with cementless hemiarthroplasty [2]. PPFs results in higher readmission rates, inferior functional outcome, and increased mortality [3,4], and high financial burden [3]. Several risk factors for PPFs have been identified, including implant design [5], bone quality [6], and implant position [7].

However, studies on the incidence and risk factors of PPFs after cementless hemiarthroplasty for FNFs are lacking. We aimed to identify the risk factors for sustaining early PPFs following hemiarthroplasty with radiological measurements.

## Materials and methods

We identified 60 consecutive patients with displaced intracapsular FNFs who underwent hemiarthroplasty between January 2018 and December 2021 at our institution. All patients underwent bipolar hemiarthroplasty with cementless stem. This study was approved by the institutional review board of Shanghai university of medicine & health Sciences Affiliated Zhoupu hospital (ZPYYLL-2022-01). it is in full accordance with international guidelines for human research protection, such as the Declaration of Helsinki, the Belmont Report. This study only collected patient imaging data information without experiments on humans and/or the use of human tissue samples. Informed consents were obtained from all patients.The exclusion criteria were as follows: substantial neurologic or musculoskeletal disorders that would adversely affect gait or early weight-bearing after surgery, and incomplete clinical data or inappropriate or incomplete radiographs. A case of PPFs was discovered within 1 week of surgery, and the patient was excluded; an undetected intraoperative fracture might have been the precursor for this fracture. Furthermore, three patients were excluded because of incomplete radiographs. Finally, 56 patients were included in this study. This study was approved by the institutional review board of the first author’s institution, and informed consent was obtained from all patients.

### Surgical procedure

All operations were performed via a standard posterolateral approach by senior surgeons with more than 30 years of experience. All patients underwent bipolar uncemented hemiarthroplasty (MicroPort Surgical Science Inc.,Shanghai,China), and the short external rotators were repaired. Preoperative templating was not routinely performed in any of the cases.

### Recorded demographics and radiological parameters

**Fig 1:**
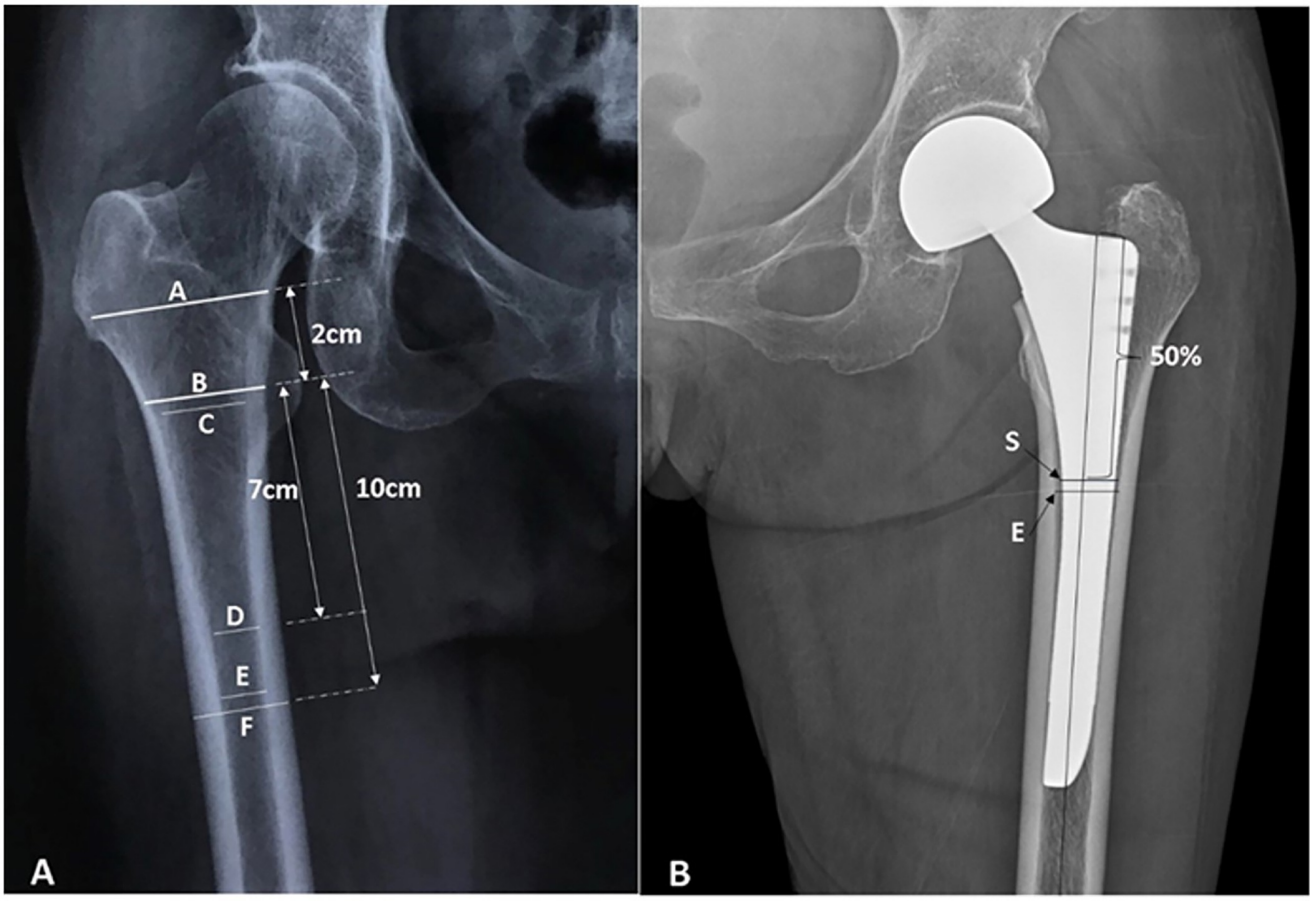
(A) Schematic diagram showing the technique used for measuring the CCR, CFI, MCI, and CBR. CCR = E/C, CFI = A/E, MCI = B/D, and CBR = E/F, as previously described [10]. (B). Schematic diagram showing the measurement of CFR (mid-third). CFR (mid-third) = S/E, where S is the stem width and E is the endosteal canal width, as previously described [12]. CCR, canal calcar ratio; CFI, canal flare index; MCI, morphologic cortical index; CBR, canal bone ratio; CFR, canal fill rate.

**Fig 2:**
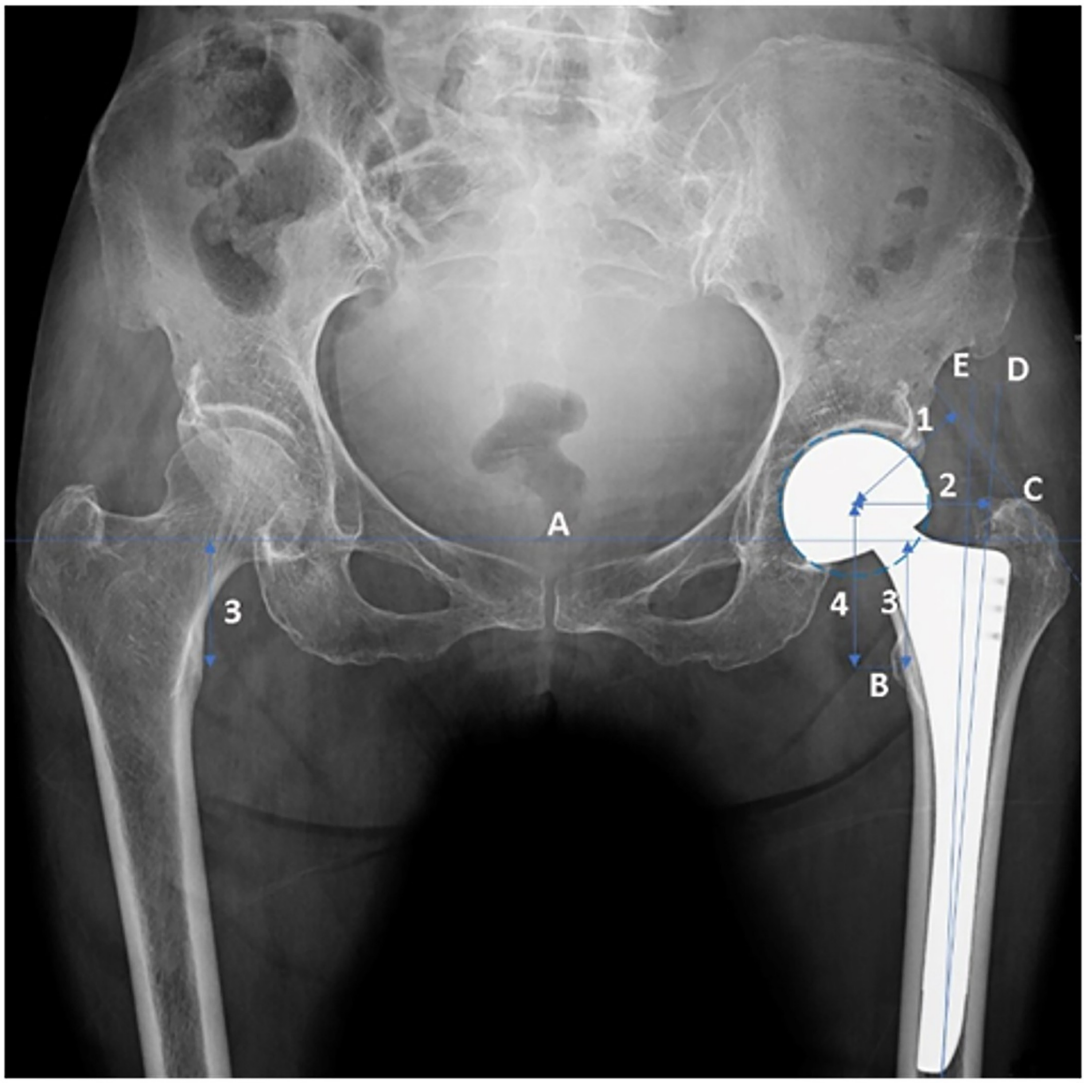
Schematic diagram showing the measurement of biomechanical parameters determined in postoperative radiographs after hemiarthroplasty: 1, abductor lever arm; 2, horizontal femoral offset; 3, limb length; and 4, vertical femoral offset. A, Horizontal tear drop line; B, midline lesser trochanter; C, tangential line to the greater trochanter; D, femoral shaft axis; and E, stem axis.

Patient characteristics, including age, sex, stem design were recorded. Femoral morphology was classified according to the Dorr type on preoperative hip radiographs as type A, B, or C [8]. Preoperative radiographic measurements performed on anteroposterior (AP) pelvis and femur radiographs included the neck-shaft angle. Measurements of the canal flare index (CFI), canal calcar ratio (CCR), morphologic cortical index (MCI), and canal bone ratio (CBR) were performed as previously described (Fig 1) [9,10]. Postoperative PPFs was classified according to the Vancouver classification system [11], and fractures were identified in medical records. For all postoperative measurements, we used the earliest postoperative image available prior to PPFs. Postoperative measurements included the stem-shaft angle, canal fill ratio (CFR), vertical and horizontal femoral offset, abductor lever arm, and leg length inequality. The varus/valgus placement of the stem, as the stem-shaft angle between the longitudinal axis of the femur and the longitudinal axis of the femoral stem on postoperative AP pelvic radiographs, was measured. The CFR was measured at 50% of the length of the stem as a ratio between the width of the implant and that of the femoral canal, as modified from a previous study [12]. Leg length is the length of a perpendicular line connecting to the trans-teardrop line; it is the most prominent point of the lesser trochanter on AP hip radiographs. The discrepancy between the leg lengths of the replaced and contralateral hips was documented as leg length inequality. Leg length inequality was measured as previously described (Fig 2) [13], using a picture archiving and communication system (GE Healthcare, Chicago, IL, USA). All patients were managed using the same comprehensive perioperative pain management and rapid rehabilitation protocol.

### Statistical analysis

Statistical analysis was performed using SPSS version 23.0 (IBM Corp., Armonk, NY, USA). Continuous variables are presented as the mean ± standard deviation. An independent samples *t* test was used to compare the two groups. Categorical variables are shown as numbers and compared using the chi-squared test or Fisher’s exact test when the expected count was lower than 5. The level of significance was set at p<0.05.

## Results

The study sample comprised 10 men and 46 women (affected hip: left, 38; right, 18). The mean patient age was 82.82±10.61 (range, 69–93) years, and the mean hemiarthroplasty to PPF time was 26.28±10.61 (range, 6.54–47.77) months. Seven (12.50%) patients had sustained PPFs (seven patients/seven hips); of these, 1 (14.29%), 5 (71.43%), and 1 (14.29%) had Vancouver classifications A, B, and C, respectively.

There were no significant differences in age, sex, affected side, Dorr classification, stem-shaft angle, CFI, MCI, CCR, abductor lever arm, or horizontal femoral offset between PPFs patients and controls (Tables 1 and 2).

**Table 1:** Study demographics of hemiarthroplasty.

Basic characteristics	PPFs patients (n = 7)	Control patients (n = 49)	*p*-Value
Age (years)	81.00 ± 7.70 (69–93)	83.10 ± 5.60 (69–92)	0.379
Sex (male/female)	3/4	7/42	0.099
Dorr (A/B/C)	1/5/1	32/14/3	0.585

Data are presented as mean ± standard deviation (range) or number of patients. Differences are considered significant at *p*<0.05. PPFs, periprosthetic fractures.

**Table 2:** Radiological outcomes of different parameters.

Parameters	PPFs patients (n = 7)	Control patients (n = 49)	*p*-Value
Canal fill ratio (%)	0.76±0.11 (0.64 to 0.95)	0.85±0.08 (0.61 to 0.99)	0.012
Canal flare index (%)	3.46±0.55 (2.63 to 4.10)	3.40±0.82 (2.30 to 6.01)	0.864
Canal calcar ratio (%)	0.69±0.10 (0.57 to 0.89)	0.69±0.12 (0.43 to 1.05)	0.996
Morphologic cortical index (%)	0.49±0.96 (0.33 to 0.61)	0.49±0.82 (0.31 to 0.63)	0.942
Stem-shaft angle (°)	−1.03±1.49 (−2.42 to 1.75)	0.28±1.77 (−3.09 to 4.33)	0.066
Cortical bone ratio (%)	0.51±0.10 (0.39 to 0.67)	0.51±0.08 (0.37 to 0.69)	0.942
Leg length inequality (mm)	−3.60±4.70 (−11.18 to 3.74)	0.63±7.73 (−16.55 to 18.89)	0.166
Abductor lever arm (mm)	−1.43±9.50 (−15.02 to 15.64)	−4.58±6.98 (−23.11 to 15.74)	0.291
Horizontal femoral offset (mm)	−7.23±8.11 (−20.88 to 2.97)	−6.79±7.86 (−24.76 to 11.19)	0.890
Vertical femoral offset	−7.57±4.87 (−14.27 to −0.96)	−2.46±6.42 (−17.28 to 14.76)	0.048

Data are presented as mean ± standard deviation (range) or number of patients. Differences are considered significant at *p*<0.05. PPFs, periprosthetic fractures.

A significant relationship was observed between the incidence of PPFs and CFR (p = 0.012); PPFs patients had a significantly smaller femoral stem CFR (0.76%±0.11%) than controls (0.85%±0.08%) (Table 2). Meanwhile, a significant shorter and unreestablished vertical femoral offset was observed in PPFs patients (p = 0.048) (Table 2).

## Discussion

Hemiarthroplasty is a recognized treatment option for the management of displaced intracapsular FNFs in frail or elderly patients, allowing for rapid recovery and mobilization and reducing the devastating risks associated with a patient being bed-bound. Despite concerns about the longevity of hemiarthroplasty, particularly for rates of acetabular wear, bipolar hemiarthroplasty has the theoretical advantage of decreasing acetabular cartilage wear and the rate of dislocation due to the dual-bearing system [14]. The optimal method for fixing the femoral stem to bone is a major controversy in THA due to varying bone quality in elderly patients. Careful consideration is necessary when selecting between press-fit and cemented stem designs. Registry data indicate an increase in the use of cementless fixation, which may seem paradoxical as these data also suggest inferior survival of cementless THA [15]. Cementless THA is associated with a higher rate of early revision and higher periprosthetic fractures (PPFs) compared to cemented THA [16]. PPFs are currently one of the leading causes of THA complications but the incidence of PPFs after hemiarthroplasty is unclear [17]. A low CFR has been consistently recognized as a risk factor for femoral stem subsidence. A ratio of less than 80% at the middle of the stem is significantly associated with an increased risk of subsidence [18]. In a study, 28.9% of patients had significant subsidence (≥5 mm), and older age and lower CFR were associated with an increased risk of implant subsidence in a study of cementless bipolar hemiarthroplasty for FNFs in elderly patients; whereas, cases with implant position A with medial overhang (x value) over the resected calcar were less prone to subsidence with the tapered proximally hydroxyapatite-coated implant. The relationship between the medial overhang of the implant and subsidence was found to be inversely related, but patients with PPFs within 12 weeks of the surgery were excluded in their study [19]. No conclusions were drawn about the relationship between PPFs, CFR, and subsidence. Kouyoumdjian et al. reported a higher PPFs rate of 10.8% in patients with a CBR >0.49, but the study lacked quantitative data on osteopenia, had 15% loss to follow-up, and did not analyze femoral stem filling [20]. Another study found that 70% of primary hip replacements were considered loose when patients sustained a PPF [21].

In this study, a significant relationship was found between PPFs and CFR (p = 0.012), with PPF patients having significantly lower CFR (0.76%±0.11%) than controls (0.85%±0.08%). Seven PPF cases were identified in the study cohort (7 patients/7 hips), and the rate of PPFs managed with an uncemented stem was deemed unacceptably high. PPFs often require further surgery, which can increase morbidity and mortality, particularly in elderly patients with coexisting medical problems [22]. The pathogenesis of PPFs is multifactorial, with surgical technique and bone quality playing important roles [23,24]. Cementless stem fixation aims for immediate stability for bony ingrowth or ongrowth, relying on a wedge fit, metaphyseal filling, or scratch fit in the diaphysis [6]. A low CFR suggests a brittle, osteoporotic femoral canal, poor bone preparation, mismatched prosthesis, and bone dimensions, or an unsuccessful press fit [25,26]; this means excessive subsidence and prosthesis instability could occur subsequently, resulting in PPFs secondary to surgical error. This was accompanied by a significant shorter and unreestablished vertical femoral offset (p = 0.048) (Table 2). Cemented stems do not require an interference fit because bone cement may serve to stabilize and anchor the prosthesis, reinforce the proximal brittle osteoporotic femur, improve load distribution, and hence, protect against the potential risk of PPFs. The underlying reasons maybe that an inexperienced orthopedic surgeon was afraid of the occurrence of intraoperative PPFs in a frail elderly patient, resulted in femoral canal with insufficient bone preparation, or a prosthetic insertion that did not in the exact position and obtain a tight press fit.

This study is limited by its retrospective design. The monocentric nature of the data and lack of randomization also presents the possibility of selection bias. The measurements were conducted on plain AP radiographs, the accuracy of which is inevitably affected by radiologic positioning and inter-observer variability. In addition, the variable magnifying effect of soft tissue could have resulted in an overestimation of the measurements. Since the focus of this study was to determine radiographic risk factors for PPFs, functional evaluation was not included, and the correlation between PPFs and clinical symptoms was not investigated. Only cementless stems were used in our patients, and there was no cement stem control; however, we compared PPF patients and controls. Finally, we did not identify cases of prosthesis instability or subsidence in our series. Four patients with Dorr type C were included, which could potentially lead to unstable stem fixation when using a cementless stem. Additionally, stem subsidence may result in a decrease in the vertical femoral offset value.

## Conclusions

A smaller femoral stem CFR is associated with a potentially high risk of PPFs in uncemented hemiarthroplasty for displaced FNFs. This may result from mismatched prosthesis and bone dimensions in the elderly population.

## Supporting information

S1 File (XLSX)
